# Supplementary material for: People choose to receive human empathy despite rating AI empathy higher
Source: Commun Psychol. 2026 Jan 31;4:19. doi: 10.1038/s44271-025-00387-3 (PMC12872445; doi:10.1038/s44271-025-00387-3)
Supplement: Supplementary file 2 — Supplemental Materials [file 44271_2025_387_MOESM2_ESM.pdf]

Contents

Complete List of Measures	2
Study 1	2
Study 2	4
Study 3	4
Study 4	5
Task Instructions	6
Study 1 and 2 Empathy Block Instructions	6
Study 1 and 2 Compassion Block Instructions	6
Study 3 Instructions	7
Study 4 Instructions	8
Study 4 Response Generation Procedure	11
Supplemental Analyses	14
Causal Inference	14
Study 1	15
Study 2	23
Study 3	31
Study 4	35
Supplemental Correlations	39
Longitudinal Analyses	42
References	44

COMPLETE LIST OF MEASURES

Study 1:

- Trial-Level Questions:
  - Visually imagine yourself in this situation. How do you feel? Write 3 words that describe your experience. (free response)
  - Choice (no explicit question, but shown a picture of two decks labeled human or AI)
  - Please evaluate the response by rating your level of agreement with the following statements. (seven-point scale, strongly disagree – strongly agree)
    - The response was empathetic
    - The response was compassionate
    - I feel connected to the responder
    - Reading this response makes me feel comforted
    - Reading this response makes me feel cared for
    - Reading this response makes me feel validated
- Post-Block Questions:
  - How did the responses from the "HUMAN" deck make you feel? (free response)
  - How did the responses from the "AI" deck make you feel? (free response)
  - How did you choose between the decks? (free response)
  - How much effort do you think the responders put into their responses from each respective deck? (five-point scale, 1 = very low, 5 = very high; asked separately for human and AI)

  - Did you develop a preference for one of the decks? (-3 [AI preference] – 3 [Human preference])
- Self Scenario
  - Participants shared a single situation from their own lives for which they wanted a caring response, then made a single choice between human and AI similar to previous choice trials.
- Interpersonal Reactivity Index, Empathic Concern subscale[1]
- Mind Perception, Experience and Agency subscales[2]
- AI Attitudes
  - AIUse: How many times have you used ChatGPT or a comparable AI in the last two weeks? (free response)
  - AIExp: How often do you use generative AI such as Chat GPT in your personal or professional life? (five-point scale, 1 = Never, 5 = All the time)
  - AIAtt: What is your general attitude towards generative AI such as ChatGPT? (-3 [Very negative] – 3 [Very positive])
  - What percentage of people tend to choose human responses in the task you just completed?
  - What percentage of people tend to choose AI responses in the task you completed?
  - AI is capable of empathy (seven-point scale, strongly disagree – strongly agree)
  - Empathy from AI is valuable (seven-point scale, strongly disagree – strongly agree)
- Demographics

    - Gender (Male, Female, Nonbinary, Other with text entry, or Prefer not to answer)
    - Ethnicity
    - Age
    - Political orientation (seven-point scale, extremely liberal – extremely conservative)
    - Loneliness (five-point scale, 1 = Never, 5 = All the time)

Study 2:

- Trial-Level Questions (same as Study 1 with following additions):
    - Visually imagine yourself in this situation. How much suffering are you experiencing? (0 [none at all] – 10 [extreme])
    - Please evaluate the response by rating your level of agreement with the following statements. (seven-point scale, strongly disagree – strongly agree)
        - The response was caring
        - The response felt authentic
    - How much effort do you think the responders put into their responses from each respective deck? (five-point scale, 1 = very low, 5 = very high)
- Post-Block Questions (same as Study 1 with removal of effort question)
- Interpersonal Reactivity Index, Empathic Concern Subscale[1]
- Mind Perception, Experience and Agency subscales[2]
- AI Attitudes (same as Study 1)
- Demographics (same as Study 1)

Study 3:

- Trial-Level Questions (same as Study 2 with removal of suffering question)

- Post-Block Questions (same as Study 2)
- Interpersonal Reactivity Index, Empathic Concern Subscale[1]
- Mind Perception, Experience and Agency subscales[2]
- AI Attitudes (same as Study 1 and 2)
- Demographics (same as Study 1 and 2)

Study 4:

- Trial-Level Questions (same as Study 3; choice measured in part 1, ratings measured in part 2)
- Post task question (How did you choose between the decks? [free response]; measured in part 1)
- Mind Perception, Experience and Agency subscales (measured in part 1)[2]
- AI Attitudes (same as Studies 1, 2, and 3; measured in part 1)
- Demographics (same as Studies 1, 2, and 3; measured in part 1)

TASK INSTRUCTIONS

Study 1 and 2 Empathy Block Instructions:

In this task, you will complete a series of trials. On each trial, you will be presented with a short story that depicts a negative event. You should read each of these stories and visually imagine yourself in the situation. You will also see two decks of cards: one deck will always be labeled “HUMAN” and the other deck will always be labeled “AI”. After reading the story, you should choose between these decks.

Once you choose a deck, you will receive an **empathetic response in which the responder will share in your experience**. The creator of this response will depend on which deck you chose. If you choose red the deck labeled, “HUMAN,” you will receive a response that was created by a human, and if you choose the blue deck labeled, “AI,” you will receive a response that was created by an AI chatbot.

You are free to choose from either deck on any trial, and should feel free to move from one deck to the other whenever you choose. If one deck begins to seem preferable, feel free to choose that deck more often. Overall, this task will take the same amount of time regardless of which deck you choose. Note also that the decks will switch sides over the course of the task. Press continue when you are ready to begin.

Study 1 and 2 Compassion Block Instructions:

In this task, you will complete a series of trials. On each trial, you will be presented with a short story that depicts a negative event. You should read each of these stories and visually imagine

yourself in the situation. You will also see two decks of cards: one deck will always be labeled "HUMAN" and the other deck will always be labeled "AI". After reading the story, you should choose between these decks.

Once you choose a deck, you will receive a **compassionate response in which the responder will express warm feelings of concern**. The creator of this response will depend on which deck you chose. If you choose red the deck labeled, "HUMAN," you will receive a response that was created by a human, and if you choose the blue deck labeled, "AI," you will receive a response that was created by an AI chatbot.

You are free to choose from either deck on any trial, and should feel free to move from one deck to the other whenever you choose. If one deck begins to seem preferable, feel free to choose that deck more often. Overall, this task will take the same amount of time regardless of which deck you choose. Note also that the decks will switch sides over the course of the task. Press continue when you are ready to begin.

Study 3 Instructions:

In this task, you will complete a series of trials. On each trial, you will be presented with a short story. You should read each of these stories and visually imagine yourself in the situation. You will also see two decks of cards: one deck will always be labeled "HUMAN" and the other deck will always be labeled "AI". After reading the story, you should choose between these decks.

Once you choose a deck, you will receive a **compassionate response**. The creator of this

response will depend on which deck you chose. If you choose red the deck labeled, “HUMAN,” you will receive a response that was created by a human, and if you choose the blue deck labeled, “AI,” you will receive a response that was created by an AI chatbot.

You are free to choose from either deck on any trial, and should feel free to move from one deck to the other whenever you choose. If one deck begins to seem preferable, feel free to choose that deck more often. Overall, this task will take the same amount of time regardless of which deck you choose. Note also that the decks will switch sides over the course of the task. Press continue when you are ready to begin.

Study 4 Instructions:

Part 1 Task Instructions:

WHAT YOU'LL DO:

In this study you will be asked to **share six different situations from your life for which you would appreciate receiving an empathetic response**—a response that expresses **warmth**, **care**, and **shares in your experience.** These situations include both negative experiences for which you could use some support and comfort, and positive experiences for which you would appreciate celebration and sharing in your joy. These situations will also be from a variety of domains including work/school, relationships, and personal struggles/triumphs. It is okay if there is overlap between categories (e.g., an experience about a work relationship), as these categories are primarily intended to help you think of different situations.

In each trial you will be prompted to **write a couple sentences describing each experience**.

Please make sure to describe the experience as if you are discussing the situation with someone. After you describe each experience, you will **choose whether you would rather receive an empathetic response created by a human or AI**.

WHAT HAPPENS AFTER:

After you complete this study, **we will select two of your six scenarios and create empathy responses from the source you chose** for each of the two scenarios. Within 24 hours, you will have the option of completing a very brief follow-up survey in which **we will return these supportive responses to you**. Your scenarios will remain completely anonymous, but please know it's okay to share only what you feel comfortable sharing.

Part 1 Scenario Prompts:

1) Think of a **positive experience** from a **relationship** (e.g., family member, friend, romantic partner). Briefly describe this situation as if you are discussing it with someone who will provide an empathetic response.
2) Think of a **positive moment** in your **work, studies, or creative efforts**. Briefly describe this situation as if you are discussing it with someone who will provide an empathetic response.
3) Think of a **positive personal situation, triumph, or high point** (e.g., can be related to mental/physical health, financial situation, a goal, etc.). Briefly describe this situation as if you are discussing it with someone who will provide an empathetic response.

4) Think of a **negative experience** from a **relationship** (e.g., family member, friend, romantic partner). Briefly describe this situation as if you are discussing it with someone who will provide an empathetic response.
5) Think of a **challenging moment** in your **work, studies, or creative efforts**. Briefly describe this situation as if you are discussing it with someone who will provide an empathetic response.
6) Think of a **negative personal situation, struggle, or low point** (e.g., can be related to mental/physical health, financial situation, a goal, etc.). Briefly describe this situation as if you are discussing it with someone who will provide an empathetic response.

Part 2 Task Instructions:

In an earlier study we asked you to share situations from your life for which you would appreciate an empathetic response, and choose whether you would like to receive a response from a human or AI. **We selected two of your six scenarios and created empathy responses from the source you chose.** The **human responses were created by other Prolific users**, and the **AI responses were created using an AI chatbot**.

In this study, **we will ask you to rate the quality of two empathy scenarios created for your specific situations**; **one response created by a human** and **one response created by an AI**. Note that if you did not choose both options in the previous study, you will only see one response instead of two.

STUDY 4 RESPONSE GENERATION PROCEDURE

Participants each described six personal scenarios in the part 1 survey. Because some participants spent substantially longer writing detailed scenario descriptions, we decided after launching to compensate participants with bonus payments depending on how long they spent on the survey. Participants were unaware of the possibility of bonus payments when signing up for and completing the survey. We compensated participants with a base payment of $3 for an estimated completion time of 15 minutes. If the participants spent 20-25 minutes we compensated them with an additional $1, if they spent 25-30 minutes we compensated them with an additional $1.50, and if they spent 30+ minutes they were compensated with an extra $2. We offered these bonus payments to participants who spent longer taking the part 1 survey primarily because they would have received a subpar hourly wage with only the base payment, and we hoped to maximize retention of participants for part 2.

As stated in the main text, following part 1, we randomly selected a scenario from each participant for which they chose human and a scenario for which they chose AI. Before generating responses, we screened all these scenarios to ensure that they were appropriate for the empathy response creation process. It was at this stage that we excluded the three participant exclusions mentioned in the main text (two for AI-use, one for writing non-scenarios). Additionally, the randomly selected human empathy scenario for one participant described childhood sexual assault. Due to the potentially triggering nature of asking another Prolific user to “share in this experience” as a part of the empathy response generation process, we decided to randomly resample from this specific participant’s other scenarios for which they chose human and use the new randomly selected scenario instead.

We then uploaded these scenarios as individual text questions within a new Qualtrics survey and used the “evenly present questions” under “advanced randomization” to show each scenario once. We then recruited a number of new participants from Prolific equal to our number of scenarios needing human responses and instructed them: “Below is a real-life scenario shared by another Prolific user for which they would appreciate receiving an empathetic response: [scenario]. Please write an empathetic response to this person in which you express **warmth**, **care**, and **share in their experience**. Your response will be shared with the original Prolific user.” Additionally, after participants wrote their empathy response, we screened them for AI use, guaranteeing that this would not affect their compensation.

Because some Prolific users began this second response creation survey then returned it without completing, the display increments for “evenly display questions” became slightly off, and we ended up with some scenarios that received multiple responses, and some scenarios without any responses. After an initial round of response creation, we filtered out the received responses from responders who admitted to using AI (based on the aforementioned screening question), and identified which scenarios did not receive any human-created responses. We adjusted our advanced randomization settings accordingly and added new participant slots on Prolific to re-recruit until we had at least one empathy response for each scenario, from a participant who specifically stated that they did not use AI (final n = 163). If certain scenarios had more than one response, we randomly selected from the received responses using R. We additionally screened this final set of responses before returning them to the part 1 participants. One of the respondents did not understand the task and instead shared a response in which they pretended they were in the original scenario alongside the scenario-writer (i.e., they made up details about other people in the scenario). This particular scenario happened to have a second

response, and because the randomly selected responder misunderstood the task, we decided to use the other empathy response instead.

As mentioned in the main text, we used the OpenAI API with ChatGPT-4o to create the AI responses. These were created by prompting the API with the following user prompt: "Please write an empathetic response to this person in which you express warmth, care, and share in their experience. Keep your response under 150 words: [scenario]". We added the word limit suggestion (though note this is just suggested in the prompt and not an actual "limit" per se) because our following survey for part 2 used a contact list with an authenticator in Qualtrics to pair participants by Prolific ID back to their original scenario and newly created response, and contact lists have a character limit for each cell. We opted for a limit suggestion in the prompt rather than restricting response length via a token-limit because restrictive token-limits can sometimes result in incomplete sentences at the end of responses once the limit is reached. We decided on a suggested limit of 150 words based on our previously generated AI response stimuli for Studies 1 and 2, as 150 words is slightly longer than the longest response from that stimulus set (which we note was created without a length cap).

Finally, once we had a complete set of responses, we uploaded these to Qualtrics as a contact list in which each row represented a part 1 participant, and we had columns for their Prolific ID, scenario in which they chose human, scenario in which they chose AI, human-generated response, and AI-generated response. We re-recruited the participants from part 1 and then used an authenticator in the part 2 Qualtrics survey to pair these participants up to their corresponding row on the contact list based on their Prolific ID, and pipe in their unique scenarios and responses. Participants were presented with their own original scenario text, the response created for their scenario, and the rating items.

SUPPLEMENTAL ANALYSES

Causal Inference

As mentioned in the main text, we ground all of our analyses (besides initial estimates derived from the paired samples t-tests reported in the supplement) in a causal inference framework. Consideration of causal inference is an essential part of the scientific process that informs which variables must be controlled for in any given analysis[3]. Common statistical approaches only show bidirectional association between variables, and it is not clear from mere statistical association whether controlling for a covariate is appropriate. For example, imagine there are two variables, X and Y, which are correlated, yet controlling for a third variable, Z, removes this association. To estimate the effect of X on Y, should we control for Z? To decide, we must consider the overall causal structure. If Z is a common cause of X and Y, the researcher should control for the common cause as the association between X and Y is spurious/noncausal. Alternatively, Z could be a mediator between X and Y (i.e., X causes Z and Z, in turn, causes Y) and controlling for it blocks true causal association between X and Y. However, Z behaves identically in the statistical analysis in both instances, so determining whether one ought to control for it is a matter of causal assumptions that do not reside in the data. There are additional cases in which controlling for a variable could even introduce spurious association that would otherwise not be present. For this reason, selecting an appropriate set of control variables (i.e., a sufficient adjustment set) to obtain unbiased estimates requires assumptions of the causal structure of variables. These assumptions are presented explicitly in the directed acyclic graphs (DAG) which we report for every study in the corresponding study section.

The DAG is informed by data, and bolstered by theory to specify the causal direction of relationships. While, in practice, some of the relational assumptions of the DAG may be incorrect and confounders may be missing, both of which threaten causal interpretation, causal

inference encourages an explicit specification of these assumptions. That is, as an additional benefit to the improved estimation of causal effects granted by appropriate modeling of controls, the transparent communication of these causal assumptions by the creation of a DAG provides a clear, concrete basis on which future researchers can disagree with and challenge our findings. This allows science to progress in a transparent, theory-driven way, rather than relying on weaker claims of association with unspecified, yet implied assumptions of causality. Put another way, instead of strategically making only associational claims, yet discussing the effect in terms of causality in which the assumptions regarding the broader causal network (i.e., possible confounders, mediators, etc.) are unspecified, causal inference requires the clear outlining of variable relationships that sets the groundwork for future research to challenge the specific assumptions made that the causal claims hinge on. Many of the principles of causal inference outlined and summarized above draw on a range of examples that can be found in most primers on the topic[4]. We encourage readers to explore the sources mentioned in the main text for introductions to the field of causal inference and a discussion of the importance of causal inference and causal graphical models for interpreting analyses and choosing appropriate control variables.

Study 1

In our DAG for Study 1 (see Figure S1), “Response Type” refers to whether the responses were empathetic or compassionate (i.e., the empathy and compassion blocks), “Suffering Type” refers to whether the vignettes depicted physical or emotional suffering, “Choice/Response Source” refers to the decision participants make to receive a response from a human or AI (when conceptualized as a dependent variable) and the source of the response they subsequently rate (when treated as an independent variable), and “Response Quality” refers to

the ratings participants make about their received response (e.g., “The response was empathetic”, “Reading this response makes me feel comforted”, etc.). Because “Response Type” and “Suffering Type” are manipulated variables, they have no causes, yet are factors that may affect whether participants choose human or AI responses, and may additionally affect response ratings if, for example, empathy responses are perceived as better than compassion responses or responses for emotional suffering are perceived as better than responses for physical suffering. Note also that any edge, such as “Choice/Response Source” -> “Response Quality”, only suggests that there is some causal relationship such that the first variable causes the latter, but does not specify the particulars of the relationship (e.g., which response source increases ratings of response quality). This type of specification is instead stated by researcher hypotheses rather than the DAG, as the DAG simply provides the set of adjustment variables which does not depend on the positive/negative direction of the causal relationship.

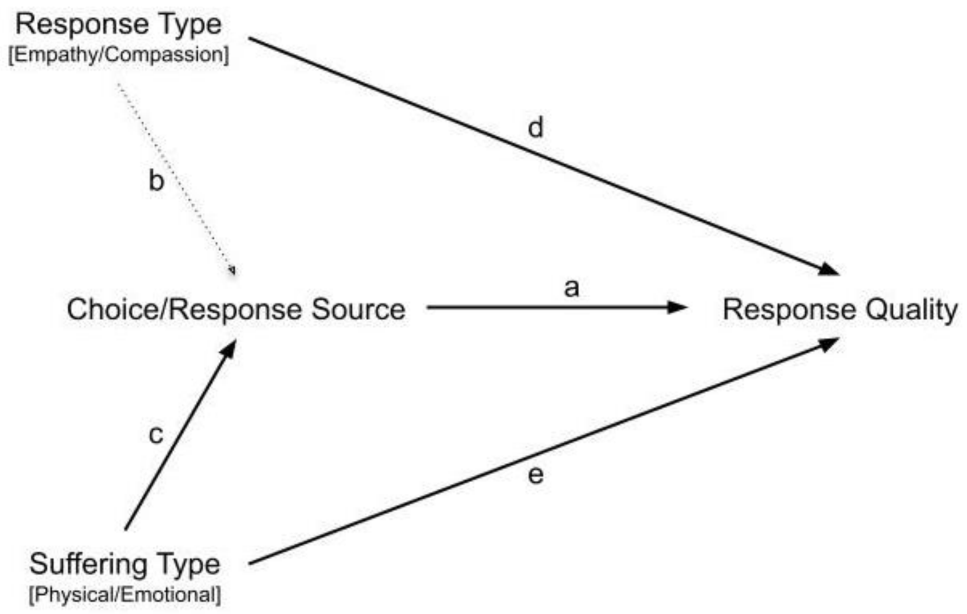

Figure S1: Study 1 DAG

The proposed directed acyclic graph (DAG) for Study 1. The dotted line indicates a path that was initially proposed, but not supported by our data.

Choice

In the choice DV logistic multilevel model reported in the main text, because logistic multilevel models do not estimate a residual within-person variance (σ^2), the ICCs for this and all subsequent logistic multilevel models were calculated using the latent threshold approach in which the residual variance is estimated to be $\frac{\pi^2}{3}$ derived from the variance of the underlying logistic distribution[5].

Empathy vs. Compassion. As can be seen in our response instructions, we differentiate empathy from compassion by its emphasis on experience-sharing, although it should be noted that this is technically a single facet of the overall construct of empathy (of which compassion could also be considered a facet). Therefore, in the context of discussing the "empathy block" and "empathy response type" we use "empathy" as shorthand for experience sharing. Additionally, we point out that the separate facets of empathy tend to commonly co-occur in everyday experiences[6]. We suggest these empathy and compassion manipulations could perhaps be taken at face value rather than as a strict differentiation between empathy and compassion, which may not be completely dissociable in the perception of empathy recipients. Instead, this can be viewed as a manipulation in which an empathizer is prompted to focus on being compassionate (i.e., generating warm feelings of concern) versus empathetic (i.e., experience-sharing) though elements of each may be present in the empathizer's responding.

We additionally mention that our instructions for the empathy responses are phrased as "an empathetic response in which you share in the experience of the person," though in many definitions of empathy it is the emotional experience, specifically, that is being shared. This distinction between sharing in the general experience versus the emotional experience may be murkier in some cases within the broader study of empathy. For example, a situation in which a

person imagines themselves feeling the physical pain and suffering of another may blur the lines between the emotion versus literal experience (as we have in our stimuli[7]). Further, while the identification of the response as an "*empathetic* response" in the instructions may nevertheless imply emotional experience sharing, AI can be sensitive to minor prompting differences, so we raise this as a possible additional consideration in the interpretation of this manipulation.

We analyzed block as a contrast-coded predictor (empathy = -0.5, compassion = 0.5) and found no difference in choice preference between the empathy and compassion blocks, $b = -0.09$, $z = -1.17$, $p = .24$, 95% CI [-0.24, 0.06], failing to provide evidence for the existence of path b in Figure S1.

Physical vs. Emotional Suffering. After this, we examined whether choice preference differed between vignettes depicting emotional suffering and vignettes depicting physical suffering (contrast code: physical suffering = -0.5, emotional suffering = 0.5). We found the odds of choosing human responses was 1.19 times higher for vignettes depicting emotional suffering relative to physical suffering (59.75% probability of choosing human for emotional suffering versus 55.49% for physical), $z = 2.31$, $p = .021$, 95% CI [0.03, 0.32], supporting the existence of path c in Figure S1. There was no evidence of an interaction between suffering type and empathy/compassion, $z = -0.51$, $p = .61$, 95% CI [-0.37, 0.22].

Response Quality

Paired Samples T-Test. We conducted an initial paired samples t-test on composite ratings that were first aggregated within participants, computing each participant's mean rating for human responses and mean rating for AI responses across all trials (collapsing across blocks). We analyzed in this way because participants rate responses from the source corresponding to their previous choice, so without first aggregating within each person, different participants have

different numbers of ratings for each source. Averaging within each person allows us to weight each participant's evaluation of human and AI responses equally, regardless of which they selected more. The paired samples t-test suggested that participants rate AI responses as significantly higher quality than human responses, $b = -0.88$, $t(146) = -9.16$, $p < .001$, $\eta_p^2 = 0.37$, 95% CI [-1.06, -0.69] (see Table S1 for means). This approach grants an initial simple and easily interpretable estimate of the within-person effect of response source, though we emphasize the multilevel modeling approach in the main text as our focal analysis as it does not require aggregation of the trial-level data, and appropriately adjusts for our causal structure depicted in the DAG.

Table S1: Descriptive statistics for Study 1

Descriptives for the Study 1 response quality composite rating calculated by averaging within each person for human and for AI choices separately, then calculating based on these aggregate ratings between people. Descriptives for effort ratings did not require this as it was natively measured at the participant level for human and AI separately.

	Human<br>*M(SD)*	AI<br>*M(SD)*
Composite Rating (1-7)	4.65 (0.92)	5.52 (0.97)
Effort (1-5)	3.15 (1.11)	3.70 (1.28)

Multilevel Model. For our centered-within-context (i.e., person-mean-centered) predictor of response source used in the main text and throughout multilevel models, we first converted response source into a contrast code (AI = -0.5, human = 0.5) then subtracted each person's mean contrast code from their original contrast code[8]. Centering-within-context is essential for

estimating within-person effects of predictors that contain a combination of between- and within-person variance, as the centering procedure disentangles these two distinct sources of variance[9].

The sufficient adjustment set for the primary analysis of response source reported in the main text consisted of a contrast-coded predictor for physical and emotional suffering (physical suffering = -0.5, emotional suffering = 0.5). This was included in the model alongside our primary predictor of response source because, assuming path c and path e exist in the DAG, there is a back-door (i.e., non-causal) pathway from "Choice/Response Source" to "Response Quality" going through "Suffering Type" which biases our estimate of the causal effect of response source (see here[10] for more in-depth explanation of the back-door criterion). Note that our previous analyses indicated a lack of evidence for path b, so there was no need to control for "Response Type."

Physical vs. Emotional Suffering. We found that participants rated responses for emotional suffering as significantly higher quality than responses for physical suffering, although this difference was modest, $b = 0.12$, $t(2887) = 2.45$, $p = .015$, $\sim R^2 < 0.01$, 95% CI [0.02, 0.21]. We also found a significant interaction between response source and suffering type, $t(2824.76) = -2.05$, $p = .040$, 95% CI [-0.38, -0.01], such that the difference between human and AI response quality ratings was greater for emotional suffering than it was for physical suffering (see Figure S2).

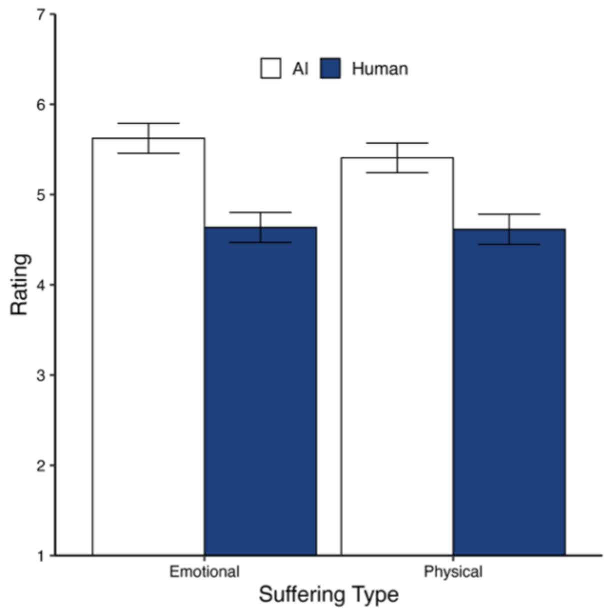

Figure S2: Study 1 estimated marginal means of composite rating split by suffering type

Plot displays the model-implied estimated marginal means for the interaction between source and suffering type for Study 1. The number of observations for AI (white) and emotional suffering is n = 622, for human (blue) and emotional suffering is n = 898, for AI and physical suffering is n = 683, and for human and physical suffering is n = 837. Error bars depict 95% confidence intervals.

Empathy vs. Compassion. Next, we tested whether there was a difference in ratings between empathy and compassion responses (path d) using a contrast-coded predictor for empathy and compassion (empathy = -0.5, compassion = 0.5) and found a modest but statistically significant effect that compassion responses were rated higher than empathy responses, supporting the existence of path d, $b = 0.12$, t(2887) = 2.54, $p = .011$, $\sim R^2 < 0.01$, 95% CI [0.03, 0.22]. We also found a significant interaction between response source and response type, $t(2795.99) = -5.19$, $p < .001$, 95% CI [-0.67, -0.30], such that the difference between human and AI response quality ratings was greater for compassion responses than it was for empathy responses (see Figure S3).

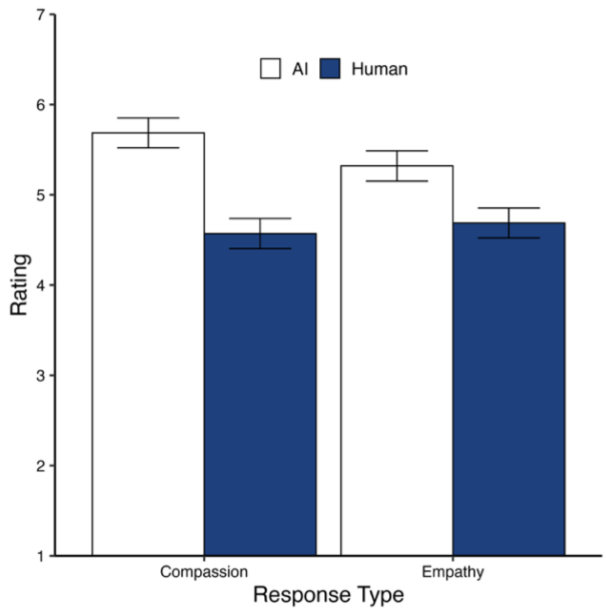

Figure S3: Study 1 estimated marginal means of composite rating split by response type

Plot displays the model-implied estimated marginal means for the interaction between source and response type for Study 1. The number of observations for AI (white) and compassion is n = 668, for human (blue) and compassion is n = 852, for AI and empathy is n = 637, and for human and empathy is n = 883. Error bars depict 95% confidence intervals.

Effort

In the main text analysis and supplemental analysis of effort below, we excluded effort ratings for individuals that did not select AI in any of the trials of the corresponding block, as they did not see AI responses and therefore could not judge the perceived effort of the responses. All participants selected human in at least one trial per block, so we did not exclude any participants based on lack of human choice. We found that empathy responses were viewed as significantly more effortful than compassion responses, $b = -0.12$, $t(138) = -2.07$, $p = .041$, $\eta_p^2 = 0.03$, 95% CI [-0.23, -0.01]. There was also a significant interaction between response type and source, $t(138) = -2.80$, $p = .006$, $\eta_p^2 = 0.05$, 95% CI [-0.33, -0.06], such that AI were perceived as putting significantly more effort into both empathy, $b = -0.42$, $t(139) = -2.80$, $p = .006$, $\eta_p^2 =$

0.05, 95% CI [-0.72, -0.12], and compassion responses $b = -0.72$, $t(145) = -4.90$, $p < .001$, $\eta_p^2 = 0.14$, 95% CI [-1.01, -0.43], with a greater difference for compassion.

Study 2

Figure S4 depicts our initial DAG for Study 2. We incorporated additional potential paths related to the effort (now measured on a trial-level) and suffering intensity measures. Additionally, in our analyses, our "Response Quality" rating variable is split into two factors: "Empathy" and "Feeling Heard." We hypothesized identical causal structures for both of these dependent variables, so to simplify the DAG we re-used "Response Quality" to refer to both of these. An explanation and justification of this rating split can be seen in the relevant Confirmatory Factor Analysis section in the main text.

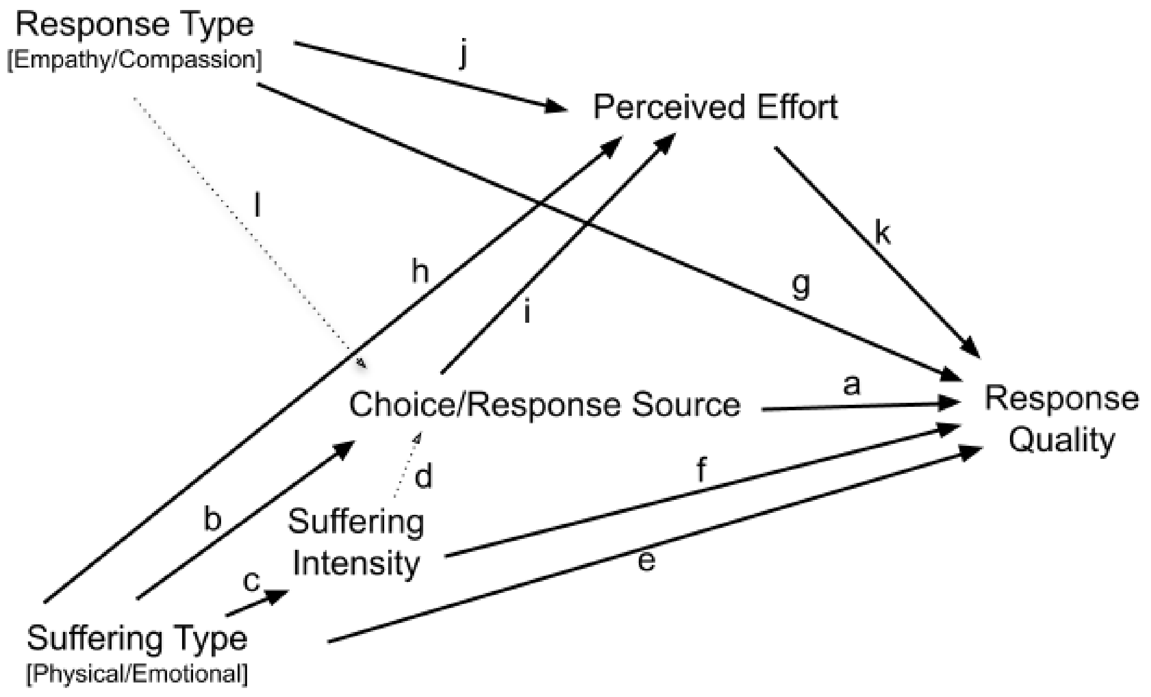

Figure S4: Study 2 DAG

The proposed directed acyclic graph (DAG) for Study 2. The dotted line indicates a path that was initially proposed, but not supported by our data.

Choice

Empathy vs. Compassion. We planned to use logistic multilevel modeling with choice as a dependent variable, however, as stated in the main text, the ICC was low (0.01), indicating that only one percent of the variance in choice existed between participants. Additionally, a random intercept did not significantly improve model fit (p = .08). This could reflect the greater homogeneity of the sample for Study 2 relative to Study 1. Because of this, we proceeded with traditional logistic regression. We first tested for a difference in choice between empathy and compassion blocks (path l in our DAG) and found no effect, replicating Study 1, b = 0.03, z = 0.42, p = .67, 95% CI [-0.10, 0.15].

Physical vs. Emotional Suffering. We then explored the relationship between suffering type and choice (path b) and, replicating our findings from the previous study, found that the odds of choosing human was approximately 1.24 times higher for emotional than physical suffering (60.41% probability of choosing human for emotional suffering versus 55.26% for physical), z = 3.27, p = .001, 95% CI [0.08, 0.34]. We found no evidence of an interaction between suffering type and empathy/compassion, z = -0.41, p = .68, 95% CI [-0.31, 0.20].

Suffering Intensity. Following this, we explored whether there was a difference in choice depending on suffering intensity. To analyze this, we controlled for physical/emotional suffering to close the back-door path shown in our proposed DAG (“Suffering Intensity” <- “Suffering Type” -> “Choice/Response Source”), and found that suffering intensity did not predict choice, b = 0.01, z = 1.01, p = .31, 95% CI [-0.01, 0.04]. Therefore, the proposed exploratory link “Suffering Intensity” -> “Choice/Response Source” (path d) was not supported.

Response Quality

We note here that the correlation between our two separate analyzed factors ("Empathy" and "Feeling Heard") was high ($r = 0.94$), suggesting that aggregating into a single factor and analyzing as we did in Study 1 may yield relatively similar results. However, the high correlation does not necessarily imply that these are not distinct constructs. Although we pre-registered that we would analyze both the single composite factor and the two separate factors, we found that the two-factor model had better model fit. Given the superior fit of the two-factor model, coupled with our a priori reasoning regarding the distinctness of the constructs in the two-factor model, we determined that analyzing the overall single factor would not provide incrementally useful information beyond the two separate factors. For these reasons, we analyzed only our pre-registered two factors.

Paired Samples T-Test. We began with our paired samples t-test approach, first aggregating each composite rating within each participant, then comparing their average human ratings to average AI ratings. Supporting our second hypothesis, we found that participants rated AI responses as significantly more empathetic than human responses, $b = -0.81$, $t(195) = -10.93$, $p < .001$, $\eta_p^2 = 0.38$, 95% CI [-0.95, -0.66], and that they made them feel significantly more heard, $b = -0.82$, $t(195) = -9.54$, $p < .001$, $\eta_p^2 = 0.32$, 95% CI [-0.99, -0.65] (see Table S2 for means).

Table S2: Descriptive statistics for Study 2

Descriptives for Study 2 calculated by averaging within each person for human and for AI choices separately, then calculating based on these aggregate ratings between people.

	Human *M(SD)*	AI *M(SD)*
Empathy (1-7)	5.01 (0.80)	5.82 (0.93)
Feeling Heard (1-7)	4.78 (0.86)	5.59 (1.08)
Effort (1-5)	3.01 (0.61)	3.77 (0.87)
Connection (1-7)	4.60 (0.92)	5.21 (1.32)
Authenticity (1-7)	4.90 (0.89)	5.35 (1.28)

Multilevel Model. In our primary analyses of response source in the main text, controlling for physical/emotional suffering was a sufficient adjustment set for this estimate due to the lack of a causal relationship between "Suffering Intensity" and "Response Type" with "Choice/Response Source" (i.e., the absence of paths d and l; otherwise "Suffering Intensity" and "Response Type" would also be a part of this adjustment set).

Physical vs. Emotional Suffering. In line with Study 1, we found evidence for a modest effect of suffering type on empathy and feeling heard such that responses for emotional suffering were rated as more empathetic, $b = 0.24$, $t(3720.80) = 5.92$, $p < .001$, $\sim R^2 = 0.01$, 95% CI [0.16, 0.32], and making participants feel more heard, $b = 0.20$, $t(3722.03) = 4.31$, $p < .001$, $\sim R^2 = 0.01$, 95% CI [0.11, 0.28]. We also found evidence of an interaction between response source and suffering type following the same pattern as Study 1 for both empathy, $t(3609.55) = -2.57$, $p = .010$ 95% CI [-0.36, -0.05], and feeling heard $t(3606.60) = -4.70$, $p < .001$, 95% CI [-0.58, -0.24],

such that the differences between human and AI in ratings of empathy and feeling heard were greater for emotional suffering than they were for physical suffering (see Figure S5).

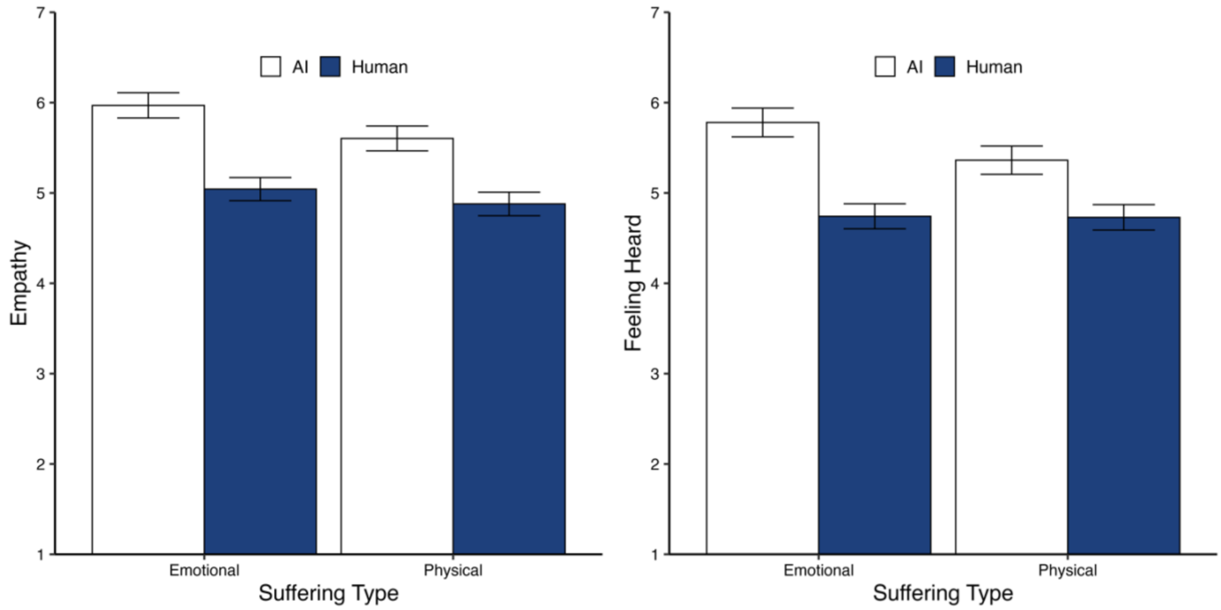

Figure S5: Study 2 estimated marginal means of Empathy and Feeling Heard split by suffering type

Plot displays the model-implied estimated marginal means for the interaction between source and suffering type for Study 2. The number of observations in the Empathy graph (left) for AI (white) and emotional suffering is n = 775, for human (blue) and emotional suffering is n = 1184, for AI and physical suffering is n = 876, and for human and physical suffering is n = 1083. The number of observations in the Feeling Heard graph (right) for AI and emotional suffering is n = 776, for human and emotional suffering is n = 1184, for AI and physical suffering is n = 877, and for human and physical suffering is n = 1082. Error bars depict 95% confidence intervals.

Empathy vs. Compassion. Next, we tested whether there was a difference in ratings of empathy (our composite factor) and/or feeling heard between empathy and compassion responses. We found small but statistically significant effect in the opposite direction of Study 1

such that compassion responses were rated lower in empathy than empathy responses, $b = -0.12$, $t(3720.87) = -2.92$, $p = .003$, $\sim R^2 < 0.01$, 95% CI [-0.20, -0.04], and lower in feeling heard, $b = -0.10$, $t(3722.03) = -2.18$, $p = .029$, $\sim R^2 < 0.01$, 95% CI [-0.19, -0.01]. This was qualified by an interaction between response source and response type for both empathy, $t(3643.68) = -6.02$, $p < .001$, 95% CI [-0.63, -0.32], and feeling heard, $t(3638.57) = -5.38$, $p < .001$, 95% CI [-0.64, -0.30], such that the differences between human and AI in ratings of empathy and feeling heard were greater for compassion than for empathy responses, replicating the interaction from Study 1 (see Figure S6).

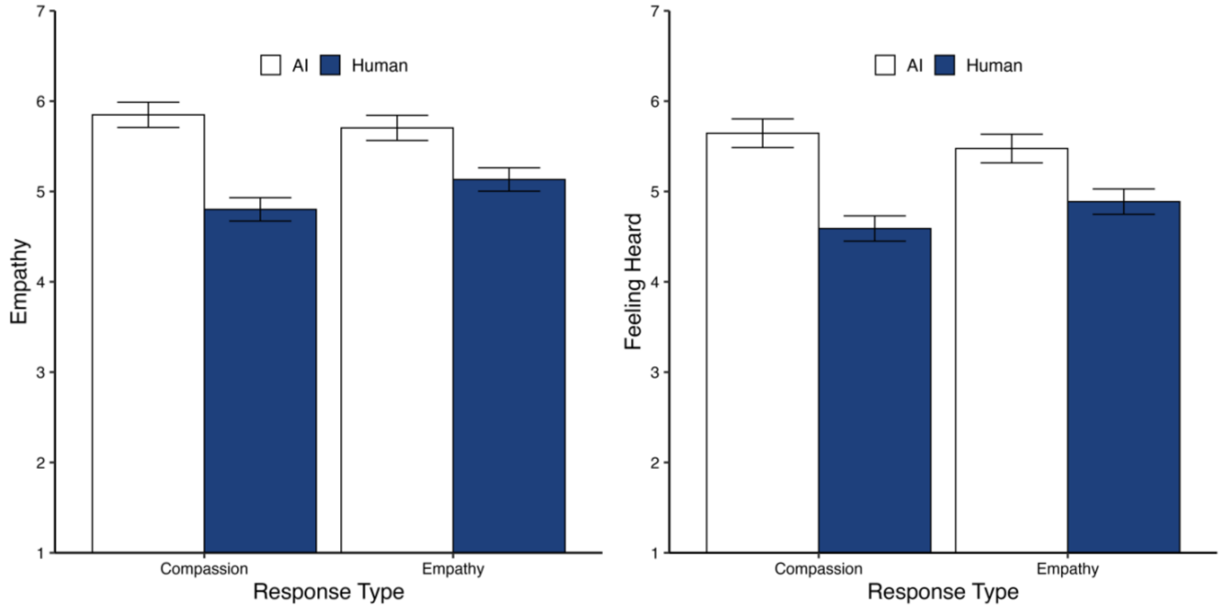

Figure S6: Study 2 estimated marginal means of Empathy and Feeling Heard split by response type

Plot displays the model-implied estimated marginal means for the interaction between source and response type for Study 2. The number of observations in the Empathy graph (left) for AI (white) and compassion is n = 820, for human (blue) and compassion is n = 1140, for AI and empathy is n = 831, and for human and empathy is n = 1127. The number of observations in the Feeling Heard graph (right) for AI and compassion is n = 820, for human and compassion is n =

1139, for AI and empathy is n = 833, and for human and empathy is n = 1127. Error bars depict 95% confidence intervals.

Suffering Intensity. We additionally found a small but statistically significant interaction between suffering intensity and response source for both empathy, $t(3570.46) = -4.33$, $p < .001$, 95% CI [-0.12, -0.05], and feeling heard, $t(3565.78) = -5.63$, $p < .001$, 95% CI = [-0.16, -0.08]. Suffering intensity had a slight negative association with ratings of response quality for human (empathy: $b = -0.06$, $t(3550) = -4.82$, $p < .001$, 95% CI [-0.09, -0.04]; feeling heard: $b = -0.11$, $t(3551) = -7.37$, $p < .001$, 95% CI [-0.14, -0.08]), but not AI sources (empathy: $b = 0.02$, $t(3595) = 1.39$, $p = .16$, 95% CI [-0.01, 0.05]; feeling heard: $b = 0.01$, $t(3584) = 0.76$, $p = .45$, 95% CI [-0.02, 0.04]). This supports the existence of path f, however the effect was quite modest so we caution against overinterpretation.

Exploratory Ratings of Connection and Authenticity

We additionally analyzed connectedness to the responder and perceived authenticity of response as additional exploratory dependent variables. We explored both in the context of multilevel models and assumed an identical causal structure as the other rating dependent variables. Random intercepts were significant for both connectedness and authenticity (p's < .001), and their ICC's = 0.21 and 0.18 respectively. Participants felt significantly more connected to AI respondents, $b = -0.63$, $t(195.16) = -6.32$, $p < .001$, $\sim R^2 = 0.20$, 95% CI [-0.83, -0.44], and found AI responses significantly more authentic, $b = -0.48$, $t(193.78) = -4.70$, $p < .001$, $\sim R^2 = 0.20$, 95% CI [-0.68, -0.28] (see Table S2 for means).

Effort

Physical vs. Emotional Suffering. The effect of suffering type on perceived effort was also significant such that responses to emotional suffering were viewed as significantly more

effortful, $b = 0.31$, $t(3717.10) = 9.13$, $p < .001$, $\sim R^2 = 0.02$, 95% CI [0.25, 0.38]. There was also a significant interaction between suffering type and response source, $t(3593.77) = -2.68$, $p = .007$, 95% CI [-0.29, -0.05], such that the human-AI gap in perceived effort was greater for emotional than physical suffering (see Figure S7).

Empathy vs. Compassion. Replicating Study 1, we found that empathy responses were viewed as having significantly more effort invested by the responder than compassion responses (path j), $b = -0.19$, $t(3717.18) = -5.52$, $p < .001$, $\sim R^2 = 0.01$, 95% CI [-0.26, -0.12]. There was also a significant interaction between response type and response source following a similar pattern as Study 1, $t(3621.67) = -6.69$, $p < .001$, 95% CI [-0.55, -0.30] (see Figure S7).

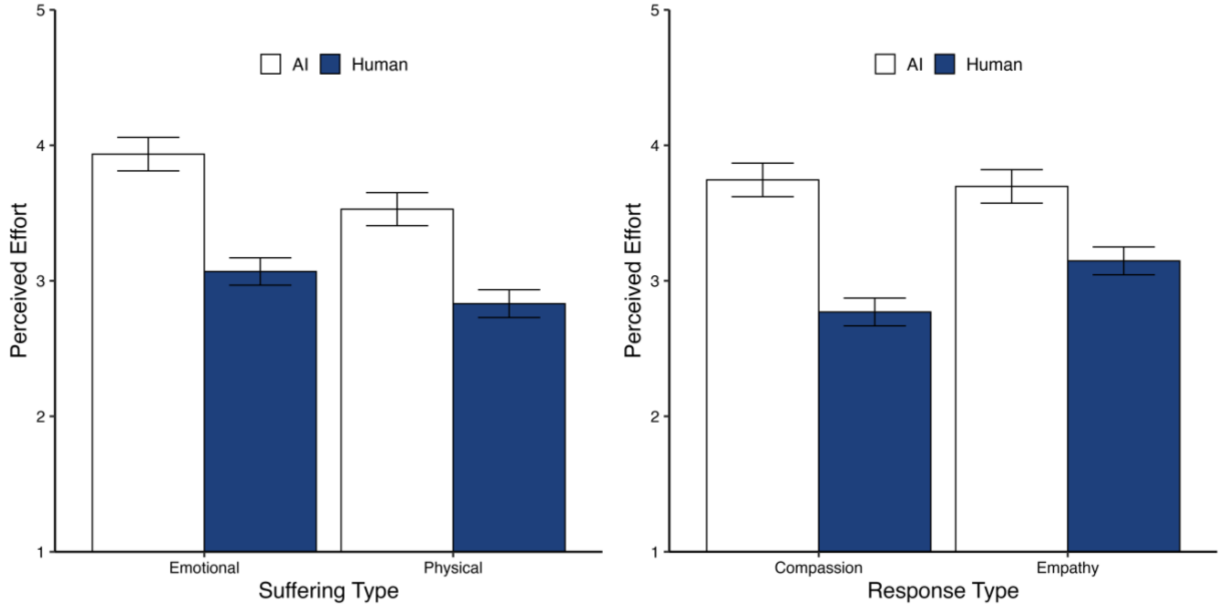

Figure S7: Study 2 estimated marginal means of Perceived Effort split by suffering type and response type

Plot displays the model-implied estimated marginal means for the interaction between source and suffering type, and source and response type for Study 2. The number of observations in the suffering type graph (left) for AI (white) and emotional suffering is n = 775, for human (blue) and emotional suffering is n = 1183, for AI and physical suffering is n = 875, and for human and

physical suffering is n = 1081. The number of observations in the response type graph (right) for AI and compassion is n = 819, for human and compassion is n = 1139, for AI and empathy is n = 831, and for human and empathy is n = 1125. Error bars depict 95% confidence intervals.

Study 3

Our updated proposed DAG for Study 3 can be seen in Figure S8. Notice the removal of "Response Type [Empathy/Compassion]," "Suffering Type [Physical/Emotional]," and "Suffering Intensity," with the addition of "Vignette Valence [Positive/Negative]" which refers to whether the vignettes depicted a positive or negative event. We ask the same response quality rating measures, and therefore analyze "Response Quality" in the same way as the previous study.

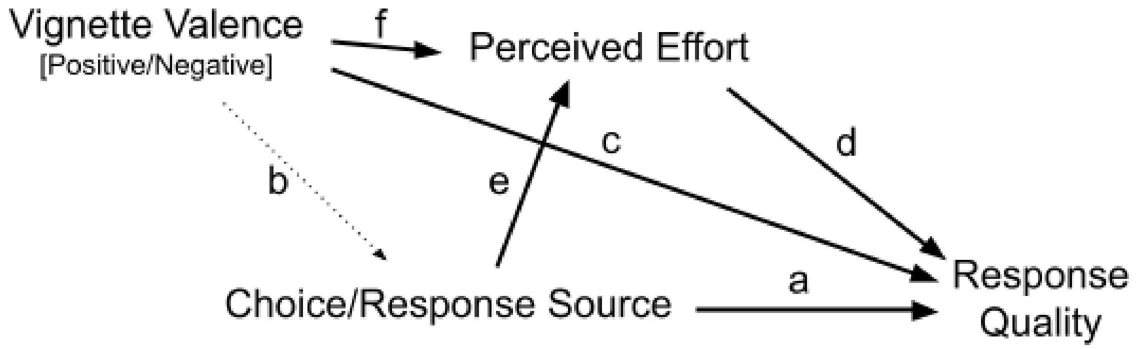

Figure S8: Study 3 DAG

The proposed directed acyclic graph (DAG) for Study 3. The dotted line indicates a path that was initially proposed, but not supported by our data.

Choice

Positive vs. Negative Valence. We analyzed the effect of vignette valence on choice (path b) using a contrast-coded predictor (negative = -0.5, positive = 0.5) and did not find a significant relationship, indicating a lack of evidence for the existence of path b, $b = 0.02$, $z = 0.31$, $p = 0.76$, 95% CI [-0.13, 0.17].

Response Quality

We note again that our two constructs, "Empathy" and "Feeling Heard," were highly correlated ($r = 0.92$). Much like Study 2, although we pre-registered that we would additionally analyze the overall single factor, we similarly decided that this would not provide useful information beyond the two separate factors

Paired Samples T-Test. Supporting our second pre-registered hypothesis, the paired samples t-test approach found that participants rated AI responses as more empathetic than human responses, $b = -0.19$, $t(181) = -2.39$, $p = .018$, $\eta_p^2 = 0.03$, 95% CI [-0.34, -0.03], and made them feel significantly more heard, $b = -0.22$, $t(181) = -2.70$, $p = .008$, $\eta_p^2 = 0.04$, 95% CI [-0.38, -0.06] (see Table S3 for means). These differences were smaller than in previous studies, which may be due to the highly curated high-quality set of human empathy responses used in the current study.

Table S3: Descriptive statistics for Study 3

Descriptives for Study 3 calculated by averaging within each person for human and for AI choices separately, then calculating based on these aggregate ratings between people.

	Human<br>*M(SD)*	AI<br>*M(SD)*
Empathy (1-7)	5.31 (0.78)	5.48 (1.13)
Feeling Heard (1-7)	5.17 (0.83)	5.36 (1.17)
Effort (1-5)	3.12 (0.66)	3.44 (0.95)
Connection (1-7)	4.85 (0.97)	4.70 (1.48)
Authenticity (1-7)	5.09 (0.89)	4.88 (1.48)

Multilevel Model. Because the previous analyses failed to support the existence of path b, in the main text we estimated the total effect of "Choice/Response Source" on empathy and feeling heard without controlling for vignette valence. That is, an empty set was a sufficient adjustment set.

Positive vs. Negative Valence. We examined the total effect of vignette valence on ratings. We found no difference between positive and negative vignettes for empathy, $b = 0.01$, $t(2924) = 0.18$, $p = .86$, 95% CI [-0.07, 0.08], and no evidence of an interaction with response source, $t(2841.38) = -0.40$, $p = .69$, 95% CI [-0.19, 0.13]. We did find a modest effect suggesting that individuals felt more heard by responses to positive vignettes, $b = 0.10$, $t(2924) = 2.50$, $p = .013$, $\sim R^2 < .01$, 95% CI [0.02, 0.18], and this did not interact with response source, $t(2846.20) = 1.02$, $p = .31$, 95% CI [-0.08, 0.26]. In summary, there were not valence by response source interactions, suggesting that AI empathy expressions outperformed human empathy expressions on ratings of empathy and feeling heard across positive and negative contexts. The lack of interactions between response source and valence does not support our third pre-registered hypothesis.

Exploratory Ratings of Connection and Authenticity

We then analyzed our additional exploratory dependent variables: connectedness to the responder and perceived authenticity. Random intercepts were significant for both connectedness and authenticity (p's < .001), and their ICC's = 0.33 and 0.29 respectively. Interestingly, and in contrast to the previous study, there was no significant difference in connection to the responder, $b = 0.12$, $t(178.64) = 1.20$, $p = .23$, 95% CI [-0.08, 0.32], or authenticity, $b = 0.18$, $t(179.29) = 1.67$, $p = .10$, 95% CI [-0.03, 0.39]. In fact, while not significant, we saw a reversal of the mean pattern for these two particular variables (see Table S3 for means). This could suggest that these

are two particular domains in which AI may not have an advantage relative to high quality human responses, as we used in this study.

Effort

Positive vs. Negative Valence. We found evidence of path f, such that responses for negative scenarios were perceived as having more effort invested into them by the responder, b = -0.09, t(2917.84) = -2.60, p = .009, ~R^2 < .01, 95% CI [-0.16, -0.02]. There was a significant interaction between response source and vignette valence, t(2841.91) = -3.08, p = .002, 95% CI [-0.37, -0.08], suggesting that the AI advantage over human empathy on effort was stronger in positive than in negative contexts (see Figure S9).

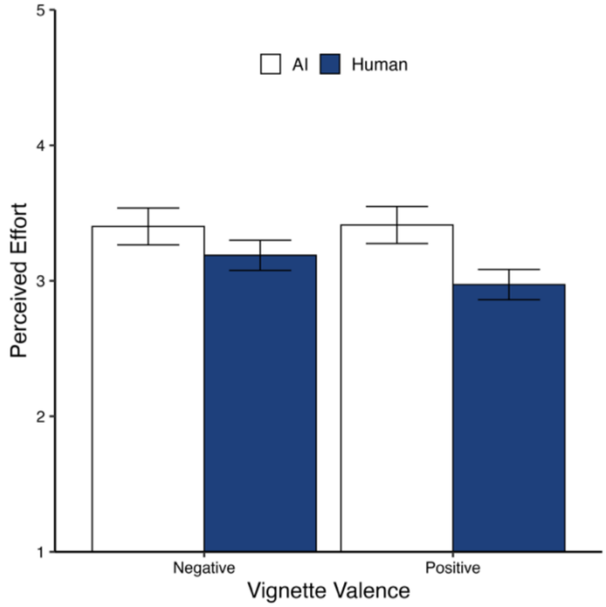

Figure S9: Study 3 estimated marginal means of Perceived Effort split by vignette valence

Plot displays the model-implied estimated marginal means for the interaction between source and vignette valence for Study 3. The number of observations for AI (white) and negative valence is n = 596, for human (blue) and negative valence is n = 961, for AI and positive valence is n = 586, and for human and positive valence is n = 971. Error bars depict 95% confidence intervals.

Study 4

Our proposed DAG for Study 4 can be seen in Figure S10. This DAG is similar to the DAG for Study 3 (Figure S8), except instead of "Vignette Valence" we have "Scenario Valence" which refers to the valence ([Positive/Negative]) that we prompted participants with as they shared their scenarios. Additionally, we have incorporated "Scenario Domain" which refers to the three different domains of prompts we gave participants as they shared their empathy scenarios. As mentioned above, these were not of particular interest, but we included them below in case they could be confounders of any of our estimates (although this turned out to not be the case). Finally, "Response Quality" refers to the same two constructs we used previously: "Empathy" and "Feeling Heard."

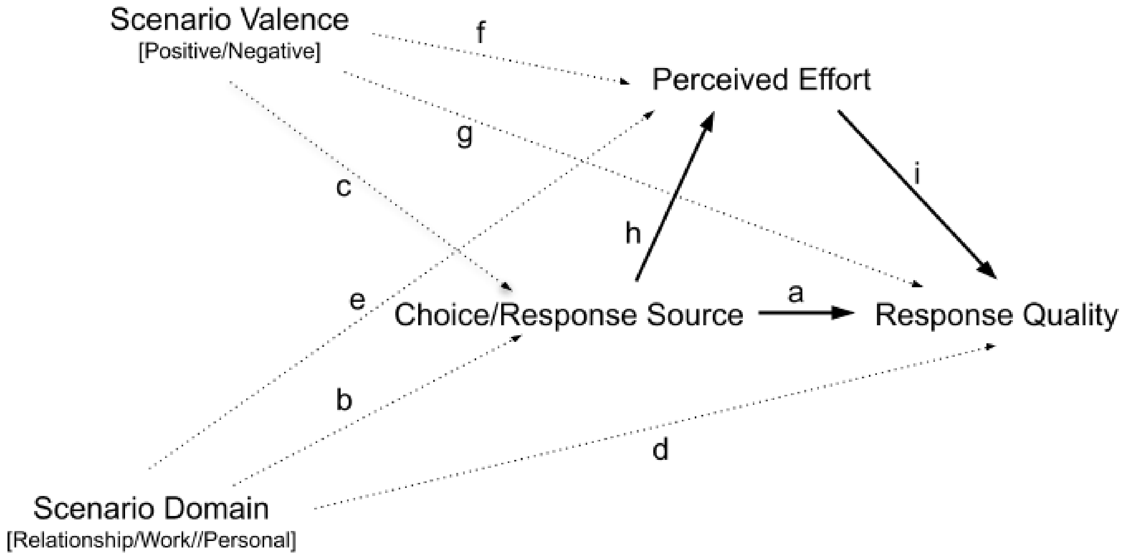

Figure S10: Study 4 DAG

The proposed directed acyclic graph (DAG) for Study 4. The dotted line indicates a path that was initially proposed, but not supported by our data.

Choice

Scenario Valence and Domain. We tested "Scenario Valence," however we found no evidence of path c, b = -0.24, z = -1.67, p = .10, 95% CI [-0.53, 0.04]. We also tested "Scenario Domain," and found that all contrasts were insignificant, z = 0.25, p = .80; z = 0.49, p = .62; z = 0.24, p = .81, indicating a lack of evidence for path b.

Response Quality

Because there was no evidence for an effect of scenario domain or valence, an empty adjustment set was sufficient. Additionally, we note that the reported paired samples t-test approach in the main text used only complete observation pairs, so participants who chose exclusively human (n = 16) or exclusively AI (n = 6) were excluded from this analysis due to the fact they would not have received a response created by a source they never chose. One could argue that this lack of choice might suggest an aversion to the responses from the unchosen source, such that their ratings may have been lower for these particular responses, had they been observed (and had they not been pleasantly surprised by the response quality). As a robustness check, we re-ran the above analysis imputing values one standard deviation below the mean response rating for these missing rating values. By imputing values one standard deviation below the corresponding mean (in contrast to a more traditional mean imputation), we intend to provide a more conservative test that assumes these participants would have disliked these responses more than the average participant, had they received them. The results of this extra robustness check completely replicate the prior findings of higher ratings of the AI responses relative to the human responses for both empathy, b = -0.24, $t(139)$ = -2.64, p = .009, η_p^2 = 0.05, 95% CI [-0.42, -0.06], and feeling heard, b = -0.24, $t(139)$ = -2.23, p = .027, η_p^2 = 0.03, 95% CI [-0.44, -0.03]. See Table S4 for means of these response ratings.

Table S4: Descriptive statistics for Study 4

Descriptives for Study 4.

	Human<br>*M(SD)*	AI<br>*M(SD)*
Empathy (1-7)	5.88 (0.92)	6.16 (0.75)
Feeling Heard (1-7)	5.78 (1.11)	6.06 (0.87)
Effort (1-5)	3.99 (1.02)	4.25 (0.89)
Connection (1-7)	5.66 (1.17)	5.86 (1.07)
Authenticity (1-7)	5.80 (1.20)	6.04 (0.98)

Scenario Valence and Domain. We also used a multilevel modeling approach for empathy and feeling heard (random intercept significantly improved model fits; ICC's = 0.23 and 0.24 respectively) to analyze our exploratory predictors of "Scenario Valence" and "Scenario Domain." Similar to our exploratory analysis with choice, none of our exploratory predictors were significant indicating a lack of evidence for paths g and d (empathy: "Scenario Valence," $t(227.09) = 0.27$, $p = .79$; "Scenario Domain," $t(224) = -0.64$, $p = .52$; $t(229) = -0.93$, $p = .35$; $t(227) = -0.27$, $p = .79$; feeling heard: "Scenario Valence," $t(223.34) = 0.59$, $p = .56$; "Scenario Domain," $t(221) = -1.16$, $p = .25$; $t(226) = -1.07$, $p = .29$; $t(224) = 0.12$, $p = .90$).

Exploratory Ratings of Connection and Authenticity

Using a paired samples t-test, we did not find a significant difference in participants' feelings of connectedness to human versus AI responders, $b = -0.17$, $t(118) = -1.34$, $p = .183$, $\eta_p^2 = 0.02$, 95% CI [-0.42, 0.08]. Similarly, we did not find a significant difference in perceived authenticity between human and AI responses, $b = -0.22$, $t(118) = -1.54$, $p = .126$, $\eta_p^2 = 0.02$,

95% CI [-0.50, 0.06]. Despite not reaching significance, AI responses were rated descriptively higher than human responses on both of these metrics. See Table S4 for means.

Effort

Scenario Valence and Domain. We used a multilevel model (significant random intercept, $p < .001$; ICC = 0.46) to analyze our exploratory predictors and again found that "Scenario Valence" ($t(194.50) = -0.11$, $p = .91$) and "Scenario Domain" ($t(189) = -0.69$, $p = .49$; $t(195) = -0.76$, $p = .45$; $t(192) = -0.06$, $p = .95$) were not significant, indicating a lack of evidence for paths f and e.

SUPPLEMENTAL CORRELATIONS

Supplemental correlation analyses are available in the supplemental code file, and results are summarized in Tables S5, S6, S7, S8, and S9. Below are the exact measures used in the calculation of those correlations.

- **Choice:** Proportion of total trials in which human is chosen
- **Response Quality, Empathy, Feeling Heard, Effort:** all difference scores calculated by averaging the corresponding rating within each participant for human and AI, then subtracting human-AI (positive indicates higher rating for human relative to AI)
- **AIAtt:** What is your general attitude towards generative AI such as ChatGPT? (-3 [Very negative] – 3 [Very positive])
- **AIUse:** How many times have you used ChatGPT or a comparable AI in the last two weeks? (free response)
- **AIExp:** How often do you use generative AI such as Chat GPT in your personal or professional life? (five-point scale, 1 = Never, 5 = All the time)
- **Experience:** possible range 1-7, higher score indicates higher attribution of experience
- **Agency:** possible range 1-7, higher score indicates higher attribution of agency

Table S5: Descriptive statistics for supplemental measures across all studies

*Supplemental descriptives; *We note that this descriptive statistic has a few highly influential observations. Though this sample seemed to have higher AI use overall, the mean is inflated. In this case, a more accurate descriptive would likely be the median, which is 10.*

	AIAtt	AIUse	AIExp	Experience	Agency
	M(SD)	*M(SD)*	*M(SD)*	*M(SD)*	*M(SD)*
Study 1	0.88 (1.64)	4.55 (7.44)	2.64 (1.05)	2.35 (1.44)	4.07 (1.50)
Study 2	0.88 (1.39)	5.82 (11.09)	2.85 (1.05)	2.57 (1.38)	4.06 (1.31)
Study 3	0.86 (1.50)	7.56 (12.98)	2.93 (1.07)	2.42 (1.30)	3.88 (1.28)
Study 4	1.91 (0.93)	28.70* (100.50)	3.75 (0.84)	3.06 (1.46)	4.60 (1.09)

Table S6: Supplemental correlations for Study 1

*Study 1 correlations; * = p < .05, ** = p < .01, *** = p < .001*

	AIAtt	AIUse	AIExp	Experience	Agency
Choice	-.29***	-.15	-.06	-.32***	-.39***
Response Quality	-.14	-.20*	-.07	-.18*	-.25**
Effort	-.24**	-.20*	-.04	-.24**	-.26**

Table S7: Supplemental correlations for Study 2

*Study 2 correlations; * = p < .05, ** = p < .01, *** = p < .001*

	AIAtt	AIUse	AIExp	Experience	Agency
Choice	-.03	.17*	.08	.05	.07
Empathy	-.04	.01	.01	-.08	-.11
Feeling Heard	-.06	.00	-.01	-.09	-.12
Effort	-.08	-.04	.02	-.11	-.18*

Table S8: Supplemental correlations for Study 3

*Study 3 correlations; * = p < .05, ** = p < .01, *** = p < .001*

	AIAtt	AIUse	AIExp	Experience	Agency
Choice	-.09	-.07	-.07	-.02	-.11
Empathy	-.15*	.00	-.06	.02	-.19**
Feeling Heard	-.21**	.01	-.09	.00	-.21**
Effort	-.13	-.04	-.03	.02	-.23**

Table S9: Supplemental correlations for Study 4

*Study 4 correlations; * = p < .05, ** = p < .01, *** = p < .001*

	AIAtt	AIUse	AIExp	Experience	Agency
Choice	-.27***	-.24**	-.30***	-.10	-.26**
Empathy	-.12	-.11	-.14	.12	.00
Feeling Heard	-.12	-.14	-.17	.13	.00
Effort	-.07	-.02	-.13	.04	-.01

LONGITUDINAL ANALYSES

The longitudinal analyses in the supplemental code file and summarized in Tables S10 and S11 examine trends in our primary outcomes across trials, split by human and AI where relevant. These analyses used a model-building approach outlined in prior work[11]. While we see a consistent descriptive trend of AI ratings increasing across trials and human ratings decreasing across trials, these effects are relatively modest with inconsistent significance. We caution against strong interpretation of these results, but we report them nonetheless. We did not conduct these analyses for Study 4 because, in part 1, participants only completed six total choice trials without receiving responses in between each choice, and they only rated two responses in part 2.

Table S10: Longitudinal analysis for Study 1

*Study 1 effects of trial order. In models with only linear effects, the coefficients represent the change in the corresponding metric per trial, but in models with both linear and quadratic effects, they represent the coefficients for polynomial contrasts. n.s. = $p \geq .05$, * = $p < .05$, ** = $p < .01$, *** = $p < .001$, N/A indicates no quadratic effect*

	Effect Type	Choice	Composite Rating AI	Composite Rating Human
Study 1	Linear	-.004, *n.s.*	.014*	-.009, *n.s.*
	Quadratic	*N/A*	-.002, *n.s.*	.002*

Table S11: Longitudinal analyses for Studies 2 and 3

*Studies 2 and 3 effects of trial order. In models with only linear effects, the coefficients represent the change in the corresponding metric per trial, but in models with both linear and quadratic effects, they represent the coefficients for polynomial contrasts. n.s. = p ≥ .05, * = p < .05, ** = p < .01, *** = p < .001, N/A indicates no quadratic effect*

	Effect Type	Choice	Empathy AI	Empathy Human	Feeling Heard AI	Feeling Heard Human
Study 2	Linear	-4.67*	.005, *n.s.*	-.017**	.018**	-.009, *n.s.*
	Quadratic	4.09*	*N/A*	*N/A*	-.001, *n.s.*	.002*
Study 3	Linear	.00, *n.s.*	.010, *n.s.*	-.010, *n.s.*	.021**	-.005, *n.s.*
	Quadratic	*N/A*	-.002, *n.s.*	.003*	*N/A*	*N/A*

Supplemental References

1. Davis, M. A Multidimensional Approach to Individual Differences in Empathy. *JSAS Cat. Sel. Doc. Psychol.* **10**, 85 (1980).
2. Gray, H. M., Gray, K. & Wegner, D. M. Dimensions of Mind Perception. *Science* **315**, 619 https://doi.org/10.1126/science.1134475 (2007).
3. Rohrer, J. M. Thinking Clearly About Correlations and Causation: Graphical Causal Models for Observational Data. *Adv. Methods Pract. Psychol. Sci.* **1**, 27–42 https://doi.org/10.1177/2515245917745629 (2018).
4. Pearl, J., Glymour, M. & Jewell, N. P. *Causal inference in statistics: A primer* (Wiley, 2016).
5. Devine, S., Uanhoro, J. O., Otto, A. R. & Flake, J. K. Approaches for Quantifying the ICC in Multilevel Logistic Models: A Didactic Demonstration. *Collabra: Psychol.* **10**, 94263 https://doi.org/10.1525/collabra.94263 (2024).
6. Depow, G. J., Francis, Z. & Inzlicht, M. The Experience of Empathy in Everyday Life. *Psychol. Sci.* **32**, 1198–1213 https://doi.org/10.1177/0956797621995202 (2021).
7. Bruneau, E., Dufour, N. & Saxe, R. How We Know It Hurts: Item Analysis of Written Narratives Reveals Distinct Neural Responses to Others' Physical Pain and Emotional Suffering. *PLoS ONE* **8**, e63085 https://doi.org/10.1371/journal.pone.0063085 (2013).
8. Yaremych, H. E., Preacher, K. J. & Hedeker, D. Centering categorical predictors in multilevel models: Best practices and interpretation. *Psychol. Methods* **28**, 613–630 https://doi.org/10.1037/met0000434 (2023).
9. Raudenbush, S. W. & Bryk, A. S. *Hierarchical Linear Models: Applications and Data Analysis Methods* (Sage, 2002).

10. Pearl, J. Causal diagrams for empirical research. Biometrika 82, 669–710 https://doi.org/10.1093/biomet/82.4.669 (1995).

11. Bliese, P. D. & Ployhart, R. E. Growth Modeling Using Random Coefficient Models: Model Building, Testing, and Illustrations. *Organ. Res. Methods* **5**, 362–387 https://doi.org/10.1177/109442802237116 (2002).
