# Supplementary material for: People choose to receive human empathy despite rating AI empathy higher
Source: Commun Psychol. 2026 Jan 31;4:19. doi: 10.1038/s44271-025-00387-3 (PMC12872445; doi:10.1038/s44271-025-00387-3)
Supplement: Supplementary file 3 — Reporting Summary [file 44271_2025_387_MOESM3_ESM.pdf]

nature portfolio

Corresponding author(s): Joshua Wenger

Last updated by author(s): Dec 12, 2025

Reporting Summary

Nature Portfolio wishes to improve the reproducibility of the work that we publish. This form provides structure for consistency and transparency in reporting. For further information on Nature Portfolio policies, see our Editorial Policies and the Editorial Policy Checklist.

Statistics

For all statistical analyses, confirm that the following items are present in the figure legend, table legend, main text, or Methods section.

n/a	Confirmed	
☐	☒	The exact sample size (*n*) for each experimental group/condition, given as a discrete number and unit of measurement
☐	☒	A statement on whether measurements were taken from distinct samples or whether the same sample was measured repeatedly
☐	☒	The statistical test(s) used AND whether they are one- or two-sided<br>*Only common tests should be described solely by name; describe more complex techniques in the Methods section.*
☐	☒	A description of all covariates tested
☐	☒	A description of any assumptions or corrections, such as tests of normality and adjustment for multiple comparisons
☐	☒	A full description of the statistical parameters including central tendency (e.g. means) or other basic estimates (e.g. regression coefficient) AND variation (e.g. standard deviation) or associated estimates of uncertainty (e.g. confidence intervals)
☐	☒	For null hypothesis testing, the test statistic (e.g. *F*, *t*, *r*) with confidence intervals, effect sizes, degrees of freedom and *P* value noted<br>*Give P values as exact values whenever suitable.*
☒	☐	For Bayesian analysis, information on the choice of priors and Markov chain Monte Carlo settings
☐	☒	For hierarchical and complex designs, identification of the appropriate level for tests and full reporting of outcomes
☐	☒	Estimates of effect sizes (e.g. Cohen's *d*, Pearson's *r*), indicating how they were calculated

Our web collection on statistics for biologists contains articles on many of the points above.

Software and code

Policy information about availability of computer code

Data collection	Data were collected using a survey created in Qualtrics and participants recruited from Prolific
Data analysis	Data were analyzed using R version 4.4.2

For manuscripts utilizing custom algorithms or software that are central to the research but not yet described in published literature, software must be made available to editors and reviewers. We strongly encourage code deposition in a community repository (e.g. GitHub). See the Nature Portfolio guidelines for submitting code & software for further information.

Data

Policy information about availability of data

All manuscripts must include a data availability statement. This statement should provide the following information, where applicable:

- Accession codes, unique identifiers, or web links for publicly available datasets
- A description of any restrictions on data availability
- For clinical datasets or third party data, please ensure that the statement adheres to our policy

All data and code are available at our Open Science Framework repository: https://osf.io/3q7ke/

Research involving human participants, their data, or biological material

Policy information about studies with human participants or human data. See also policy information about sex, gender (identity/presentation), and sexual orientation and race, ethnicity and racism.

Reporting on sex and gender	We did not analyze sex or gender as we did not have a priori predictions regarding the relevance of these predictors. We collected data on gender via self report which can be seen in the openly available data, and we report the exact gender breakdown for each study in the main text. Pooled across all studies, our sample included 494 females, 189 males, 7 nonbinary, and 1 prefer not to answer.
Reporting on race, ethnicity, or other socially relevant groupings	We did not control for race, ethnicity, or person-level factors in our analyses because all of our effects were within-person. Additionally, we did not have a priori predictions regarding the relevance of these predictors. We did collect ethnicity as a self-report variable, though have not analyzed it in any way. We report the specific per-study breakdown of ethnicity in the main text. Pooled across all studies, our sample included 432 White/Caucasian, 106 Black/African American, 40 Hispanic/Latino, 38 Asian/Pacific Islander, 2 Native American, 7 other, 1 non-response, and 65 mixed-race participants. All control variables were chosen based on a causal inference approach alluded to in the main text and outlined in detail in the supplemental materials.
Population characteristics	We did not include age as a covariate in any study, but we did collect a self-report measure of age, and we report average age and the standard deviation of age for each study in the main text. The average age for Studies 1 and 4 (pooling because both were from Prolific samples) was 37.69 years with a standard deviation of 12.75. The average age for Studies 2 and 3 (pooling because both were from undergraduate research samples) was 18.91 years with a standard deviation of 1.48.
Recruitment	Participants were recruited from a mix of the crowdsourcing platform Prolific (Studies 1 and 4) and an undergraduate research pool in the eastern United States (Studies 2 and 3).The undergraduate research pool is notably less diverse as it consists of exclusively university students, however Prolific includes a more diverse array of participants. Results were robust across both of these populations, reducing the potential issue of generalizibility.
Ethics oversight	All studies were approved by the Pennsylvania State University IRB

Note that full information on the approval of the study protocol must also be provided in the manuscript.

Field-specific reporting

Please select the one below that is the best fit for your research. If you are not sure, read the appropriate sections before making your selection.

☐ Life sciences ☒ Behavioural & social sciences ☐ Ecological, evolutionary & environmental sciences

For a reference copy of the document with all sections, see nature.com/documents/nr-reporting-summary-flat.pdf

Behavioural & social sciences study design

All studies must disclose on these points even when the disclosure is negative.

Study description	In this set of studies, participants completed a series of trials in which they chose between receiving empathy from a human or AI source. After each trial, participants received an empathic response from the source of their choosing and rated each response in terms of its empathic quality.
Research sample	In Study 1 we recruited 152 US participants using Prolific and screened for English fluency with an in-built Prolific screener. In Study 2 we recruited 196 participants from the undergraduate research pool at the Pennsylvania State University. In Study 3 we recruited 195 participants from the undergraduate research pool at the Pennsylvania State University. In Study 4 we recruited 148 US participants using Prolific and screened for English fluency with an in-built Prolific screener. Demographics of participants are described above, described for each study in the main text, and available on a per-participant basis in the openly available data.
Sampling strategy	All sample sizes were determined based on power-analyses using G*Power 3.1. In each study we powered to detect a small-medium effect for both a one sample t-test used to analyze choice, and an initial paired samples t-test (reported in the supplemental materials) to analyze response ratings. Norms in psychology recommend power of at least 80%, which we achieve in all studies. Higher power is often preferred, so Studies 2-4 additionally targeted higher power due to greater resources for participant recruitment at the time of the studies.
Data collection	Across all studies, participants signed up for and completed the online surveys using their own personal computers. Participants in Studies 1 and 4 signed up for surveys on the Prolific platform, while participants in Studies 2 and 3 signed up for surveys on the SONA platform. Participants recruited from Prolific were compensated at a rate of ~$12/hr for all studies, and participants recruited from SONA received undergraduate course credit as compensation for their participation.
Timing	Study 1 was conducted in October of 2024, Study 2 in December of 2024, Study 3 in December of 2024, and Study 4 in June of 2025.
Data exclusions	Participants were excluded based on pre-registered exclusion criteria involving missing data, copy and pasting nonsense sentence fragments in the free response boxes, or (for Study 4) disclosing their own use of AI. We excluded 1 participant in Study 1, 5 participants in Study 2, 5 participants in Study 3, and 3 participants in Study 4.

Non-participation

Participants who declined the informed consent at the very beginning of each survey were excluded and did not provide data. Study 4 was the only study that involved multiple waves of data collection (though all within a single day), and in this study 140 of the 148 participants completed the second survey.

Randomization

Participants were not assigned to between-subjects experimental conditions. Within-subject manipulation order was randomized and counterbalanced across participants. The stimulus display order was also randomized.

Reporting for specific materials, systems and methods

We require information from authors about some types of materials, experimental systems and methods used in many studies. Here, indicate whether each material, system or method listed is relevant to your study. If you are not sure if a list item applies to your research, read the appropriate section before selecting a response.

Materials & experimental systems

n/a	Involved in the study
☒	☐ Antibodies
☒	☐ Eukaryotic cell lines
☒	☐ Palaeontology and archaeology
☒	☐ Animals and other organisms
☒	☐ Clinical data
☒	☐ Dual use research of concern
☒	☐ Plants

Methods

n/a	Involved in the study
☒	☐ ChIP-seq
☒	☐ Flow cytometry
☒	☐ MRI-based neuroimaging

Plants

Seed stocks

Report on the source of all seed stocks or other plant material used. If applicable, state the seed stock centre and catalogue number. If plant specimens were collected from the field, describe the collection location, date and sampling procedures.

Novel plant genotypes

Describe the methods by which all novel plant genotypes were produced. This includes those generated by transgenic approaches, gene editing, chemical/radiation-based mutagenesis and hybridization. For transgenic lines, describe the transformation method, the number of independent lines analyzed and the generation upon which experiments were performed. For gene-edited lines, describe the editor used, the endogenous sequence targeted for editing, the targeting guide RNA sequence (if applicable) and how the editor was applied.

Authentication

Describe any authentication procedures for each seed stock used or novel genotype generated. Describe any experiments used to assess the effect of a mutation and, where applicable, how potential secondary effects (e.g. second site T-DNA insertions, mosiacism, off-target gene editing) were examined.
